# Shining a Spotlight on Gerontology: Enhancing Visibility and Impact in Aging Programs

**DOI:** 10.1093/geroni/igaf122.4009

**Published:** 2025-12-31

**Authors:** Naomi Adjei, Julie Masters, Elsa Wilcox, Mark Staley, Hailey Baca

**Affiliations:** Dartmouth College, The Dartmouth Institute for Health Policy & Clinical Practice, New Hampshire, United States; UNO, Omaha, Nebraska, United States; University of Nebraska-Lincoln, University of Nebraska-Lincoln, Nebraska, United States; University of Nebraska-Lincoln, University of Nebraska-Lincoln, Nebraska, United States; University of Nebraska-Lincoln, Lincoln, Nebraska, United States

## Abstract

Despite the rapid aging of the U.S. population and the growing demand for professionals trained to support older adults, Gerontology programs across the country have experienced a troubling decline in visibility, enrollment, and institutional support. Over the past decade, several undergraduate and graduate Gerontology programs have been downsized, merged with other disciplines, or eliminated entirely. This decline persists even as the societal need for aging-focused expertise intensifies. This study investigates the visibility and enrollment dynamics of Gerontology programs across the United States by analyzing institutional size, program-level enrollment, recruitment strategies, and disciplinary intersections. The sample included 74 colleges with Gerontology programs. Results indicate that a majority of institutions offering Gerontology programs are small to mid-sized, with 0–5,000 and 5,001–10,000 student enrollments comprising the largest segments highlighting the predominance of smaller educational settings in the field. Analysis of recruitment methods shows that college advisors (over 70%), in-class presentations (50%), and printed materials (approximately 47%) were perceived as the most successful strategies for attracting students from other disciplines. Less effective approaches included community presentations and partnerships with high schools. These findings indicate the importance of direct academic engagement of program awareness. In addition, data suggests strong interdisciplinary overlap between Gerontology and human services or behavioral health fields, emphasizing the need for cross-departmental collaboration in curriculum design and student recruitment. Together, the data highlight the opportunities and challenges facing Gerontology programs. Improving visibility through targeted advising and strategic partnerships, along with exploring interdisciplinary collaborations, may enhance program growth and sustainability.

